# HMGB1-mediated autophagy attenuates gemcitabine-induced apoptosis in bladder cancer cells involving JNK and ERK activation

**DOI:** 10.18632/oncotarget.17796

**Published:** 2017-05-11

**Authors:** Hubin Yin, Xiaoyu Yang, Wen Gu, Yan Liu, Xinyuan Li, Xiaolong Huang, Xin Zhu, Yong Tao, Xin Gou, Weiyang He

**Affiliations:** ^1^ Department of Urology, The First Affiliated Hospital of Chongqing Medical University, Chongqing 400016, China; ^2^ Chongqing Key Laboratory of Molecular Oncology and Epigenetics, The First Affiliated Hospital of Chongqing Medical University, Chongqing 400016, China

**Keywords:** high-mobility group box 1, autophagy, chemoresistance, mitogen-activated protein kinase, bladder carcinoma

## Abstract

High-mobility group box 1 (HMGB1) has been found to mediate autophagy during chemotherapy in several cancers. However, whether HMGB1plays a role in autophagy and chemoresistance in bladder cancer is elusive. In this report, HMGB1 expression was found to be increased in 30 primary bladder cancer tissue specimens compared to their matched adjacent non-tumor tissues. While gemcitabine induced apoptotic cell death, it also induced HMGB1 expression and autophagy in bladder cancer T24 and BIU-87 cells. Suppressing HMGB1 expression with siRNA strongly potentiated gemcitabine-induced apoptosis. HMGB1 siRNA or autophagy inhibitors suppressed gemcitabine-induced autophagy. Further, gemcitabine activated c-Jun N-terminal kinase (JNK) and extracellular regulated protein kinase (ERK) and Bcl-2 phosphorylation, and blocking ERK and JNK inhibited autophagy and increased apoptosis in gemcitabine-treated cells. Interestingly, suppressing HMGB1 expression attenuated gemcitabine-induced ERK and JNK activation and Bcl-2 phosphorylation. Thus, our results suggest that while gemcitabine kills bladder cancer cells through apoptosis, a cytoprotective autophagy is also induced involving HMGB1-mediated JNK and ERK to counteract the cytotoxicity of gemcitabine, and intervention targeting this pathway may improve the anticancer efficacy of gemcitabine against bladder cancer.

## INTRODUCTION

Urinary bladder carcinoma is the fifth most common type of cancer among all cancers and the second most common urologic malignancy [1]. The majority of bladder cancer patients are diagnosed as the type of non-muscle invasive tumor that has a high rate of recurrence. Almost one third of these tumors will develop into muscle invasive, metastasis and life-threatening type that requires further treatment [2, 3]. The regimen of gemcitabine (GEM) plus cisplatin is the frontier treatment, because it exerts a clinical efficacy equivalent to that of conventional chemotherapy regimens but has much lower side effects [4]. Gemcitabine, a member of pyrimidine antimetabolite, has been proved to be effective in treating bladder transitional cell carcinoma and other malignancies [5]. Unfortunately, the clinical application of gemcitabine is hampered by chemoresistance.

High mobility group box 1(HMGB1) is a highly conserved non-histone nuclear protein that is involved in damage response. During response to various exogenous and endogenous stimuli, HMGB1 is actively translocated from the nucleus to the cytoplasm or passively released to outside of the cells, where HMGB1 binds to several receptors to activate signaling pathways for promotion of cell proliferation, inhibition of apoptosis and induction of autophagy [6–8].

As a major cell component degradation and recycling mechanism, autophagy is indispensable for cell development and metabolism. Dysfunction of autophagy has been implicated in multiple pathological conditions [9]. There are paradoxical effects of autophagy in cell death in tumor, either pro- or anti-death, which depends on tumor types, stages, grades, genetic contexts and cellular microenvironments [6]. Consistently, autophagy has complicated roles in response of tumor cells to chemotherapy. It was reported gemcitabine induces autophagy induction in bladder cancer [10], however, the underlying mechanism of which is elusive.

In this study, we investigated the role and mechanism of autophagy in the response of bladder cancer cells to GEM. We found that HMGB1 is overexpressed in human bladder cancers with different stages and grades. GEM induced HMGB1 expression and autophagy activation. Suppressing HMGB1 expression attenuated autophagy and potently enhanced apoptosis in GEM-treated bladder cancer cells. Furthermore, we identified a novel pathway consists of HMGB1, ERK, JNK and Bcl-2 for GEM-induced cytoprotective autophagy, and intervention targeting this pathway may improve the anticancer activity of GEM against bladder cancer.

## RESULTS

### HMGB1 is highly expressed in bladder cancer tissues and correlated to cancer progression and clinicopathologic features

**Figure 1:**
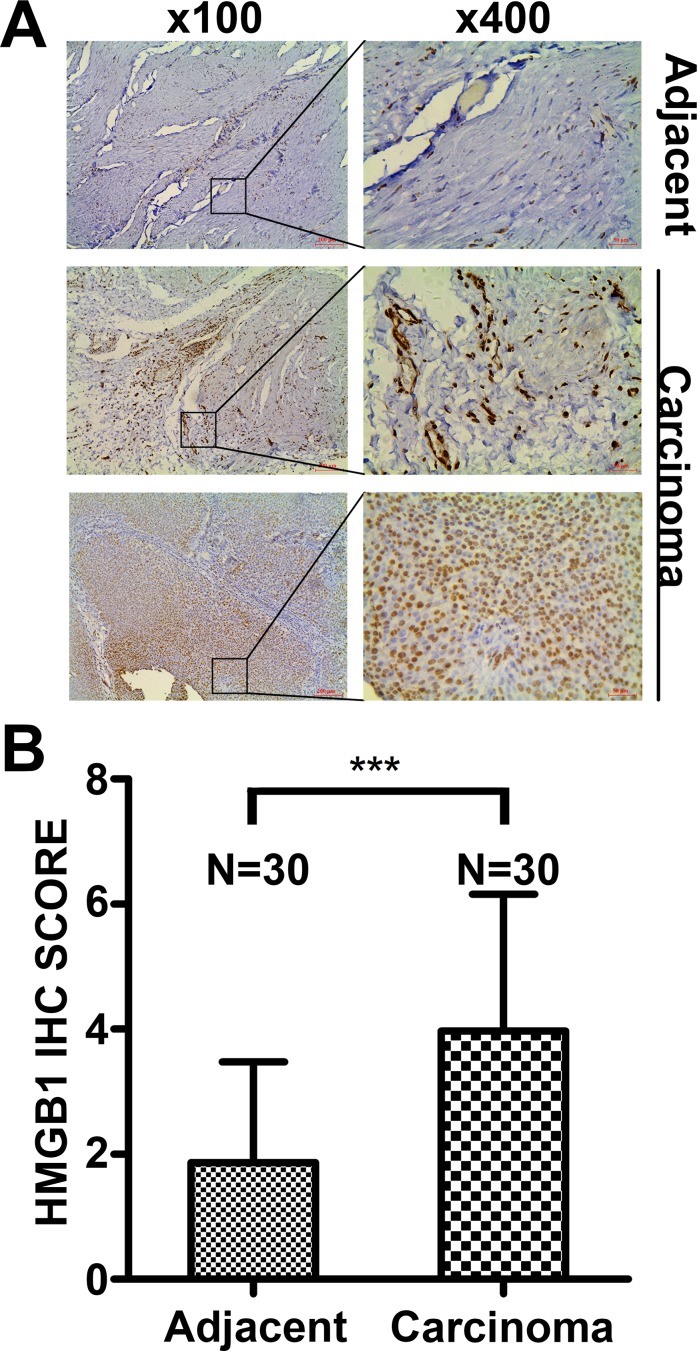
HMGB1 expression in bladder cancer tissues and adjacent non-tumor tissues detected by immunohistochemistry (**A**) Adjacent tissues: Low HMGB1 expression in the majority of adjacent non-tumor samples. Carcinoma tissues: Low HMGB1 expression in a portion of bladder cancer tissues (19/51) and high HMGB1 expression in most individuals (32/51). Scale Bar = 200 μm (Original magnification: ×100); Scale Bar = 50 μm (Original magnification: ×400). (**B**) The difference of HMGB1 IHC Score in paired normal and cancer tissues. (***p < 0.001)

Paired bladder cancer tissues and paired adjacent non-tumor tissues from thirty patients were collected and used for detecting expression by immunohistochemistry. The majority of carcinoma samples (32/51) showed a higher expression of HMGB1, while the rest (19/51) had a lower HMGB1 level. HMGB1 was detected predominantly in the nuclei, and its expression level in cancer tissues was significantly higher than that in paired adjacent non-tumor tissues (Figure 1A–1B). In addition, HMGB1 expression was closely correlated to tumor grade and T stage (*p* = 0.006 and *p* = 0.015, respectively) but not to age, gender, tumor size and number (Table 1). These results suggest HMGB1 protein expression is increased and associated with tumor grade and T stage in bladder carcinoma.

**Table 1:** Clinicopathological characteristics of HMGB1 expression in patients with bladder carcinoma

Parameter	Number	HMGB1 expression		*P* value
		Low	High	
Gender				1.000
Female	5	2	3	
Male	46	17	29	
Age (years)				0.385
≤ 65	24	7	17	
> 65	27	12	15	
Tumor size (cm)				1.000
≤ 3	23	9	14	
> 3	28	10	18	
Tumor number				0.250
Unifocal	22	6	16	
Multifocal	29	13	16	
Grade				0.006
G2	17	11	6	
G3	34	8	26	
T stage				0.015
Ta-T1	18	11	7	
T2–T4	33	8	25	

*P* values were calculated from χ^2^ test.

### Gemcitabine induces apoptotic cytotoxicity in bladder urothelial carcinoma

**Figure 2:**
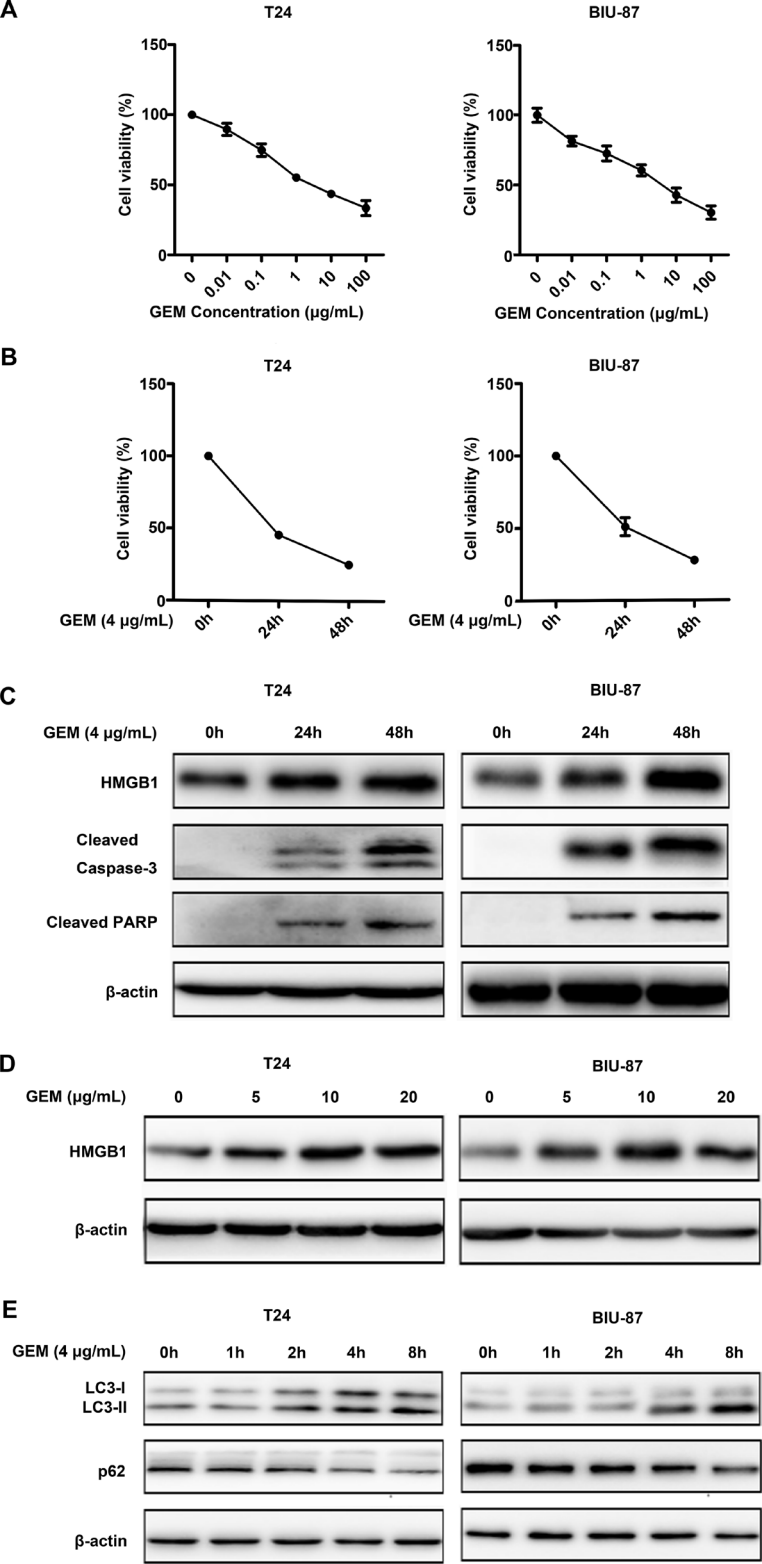
Gemcitabine induces apoptotic cell death and HMGB1 expression in bladder urothelial carcinoma cells (**A**, **B**) CCK8 assay showed GEM inhibited cell viability of T24 and BU-87 in a dose- and time-dependent manner. Dates shown are means ± standard deviation (SD) from at least three independent experiments. (**C**) The cells were treated with GEM (4 μg/mL) for the indicated time, and then cell lysates were prepared for detection of cleaved caspase-3, cleaved PARP and HMGB1 expression by Western blotting. β-actin was detected as an input control. (**D**) The cells were treated with different concentration of GEM for 24 h, HMGB1 expression was measured by Western blotting. (**E**) The cells were treated with GEM (4 μg/mL) for the indicated time, LC3 and p62 were measured by Western blotting.

In order to investigate the anticancer capability of GEM against bladder cancer cells, we treated the bladder cancer cell lines T24 and BIU-87 with a serial concentrations ranged from 0.01 to 100 μg/mL for 24 h, followed by detecting cell viability by CCK8 assay, GEM treatment significantly reduced viability decreased in a dose- and time-dependent manner in both the T24 and BIU-87 cells (Figure 2A, 2B). The half maximal inhibitory concentration (IC50) of T24 and BIU-87 was 4.3576 ± 0.8144 μ g /mL (mean ± SEM) and 4.004 ± 1.029 μg/mL (mean ± SEM) at 24 h, respectively. Therefore, the concentration of 4 μg/mL was chosen for subsequent experiments. It was previously reported that endogenous HMGB1 expression was increased after treatment with various chemotherapy drugs, which is likely associated with drug resistance [31]. Thus, we investigated whether GEM affects HMGB1 expression in bladder cancer cells. Indeed, the expression level of HMGB1 was increased in a dose- and time-dependent manner after GEM treatment (Figure 2C, 2D). Meanwhile, apoptosis was induced, which was shown as cleavage of caspase-3 and its substrate PARP (Figure 2C). Besides, HMGB1 treatment also induced autophagy, which was detected as increase of LC3-II and decrease of p62 (Figure 2E). These experiments suggest that while GEM kills bladder cancer cells through apoptosis, it also increases HMGB1 expression and induces autophagy.

### Suppression HMGB1 expression results in declined cell viability

**Figure 3:**
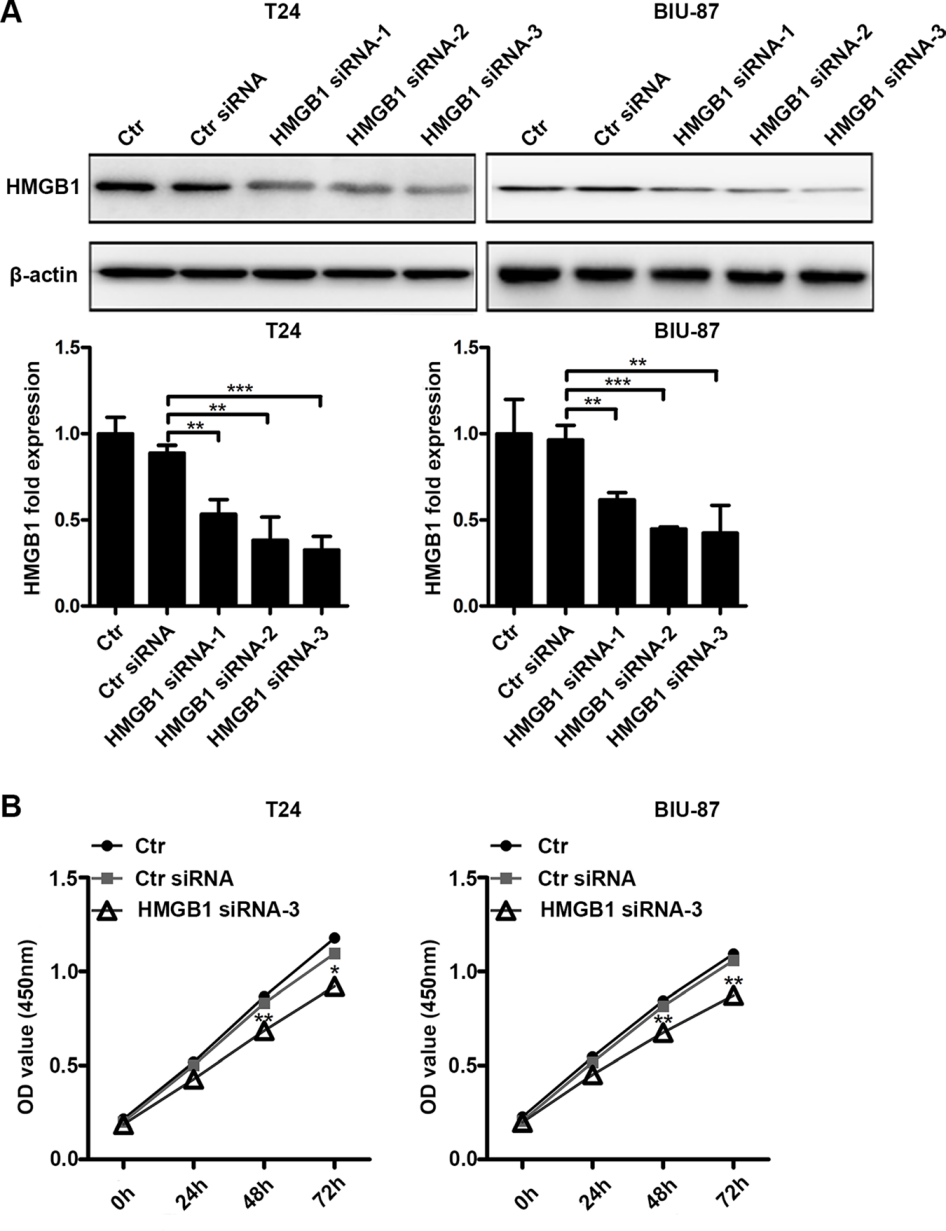
Suppressing HMGB1 expression results in declined cell viability (**A**) The cells were untreated or transfected with control siRNA or three kinds of HMGB1 siRNA (siRNA-1, siRNA-2 or siRNA-3) for 48 h, then cell lysates were prepared for testing HMGB1 protein expression by western blotting. Data are presented as mean ± SD from three independent experiments. (Ctr: Control). (**B**) The cells were untreated or transfected with control siRNA or HMGB1 siRNA-3 for indicated time periods in 96-well plate, then cell proliferation was measured by CCK8 assay. Data are shown from three independent experiments. (*p < 0.05, **p < 0.01 and ***p < 0.001 compared with control siRNA group).

We used RNAi to knock down HMGB1 expression. Of the tested siRNAs, siRNA-3 exerted the best effect in suppressing HMGB1 expression in both T24 and BIU-87 cells (Figure 3A). The siRNA-3 was chosen for the subsequent experiments. We next examined the effect of HMGB1 knockdown on cell viability detected by CCK8 assay. Compared to control groups, siRNA-3 transfection suppressed cell viability (Figure 3B), suggesting that HMGB1 is involved in cell viability in bladder cancer cells.

### HMGB1 knockdown enhances the sensitivity of bladder cancer cells to gemcitabine *in vitro*

**Figure 4:**
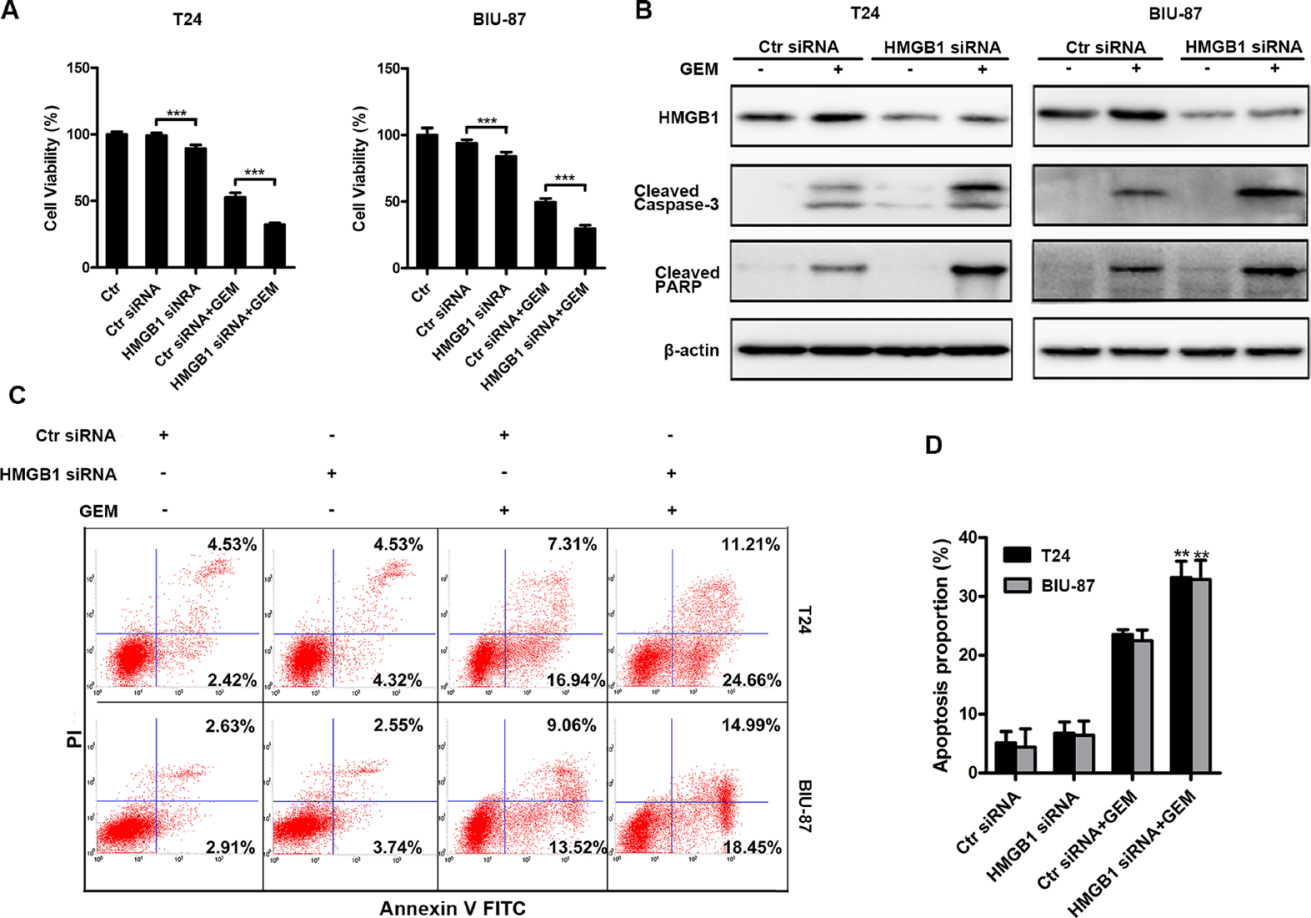
Down-regulation of HMGB1 expression enhances GEM-induced apoptosis (**A**) The indicated cells were transfected with or without control siRNA or HMGB1 siRNA for 48 h and then dealt with GEM (4 μg/mL) for a further 24 h. At the end of the treatment, cell viability was analyzed by CCK8 kit. Dates shown are the means ± SD from at least three independent experiments. (***p < 0.001). (**B**) The indicated cells were transfected with control siRNA or HMGB1 siRNA for 48 h and then treated with GEM (4 μg/mL) for another 24 h, after that whole-cell lysates were prepared for detecting cleaved caspase-3 and cleaved PARP by western blotting. (**C**) The cells were transfected with RNAi for 48 h and treated with GEM (4 μg/mL) for 24 h, respectively. Then apoptosis was measured by detecting positive percentage of Annexin V cells in flow cytometry. (**D**) The apoptosis rate was adding annexin V+/PI- (early apoptosis rate) and annexin V+/PI+ (late apoptosis rate). Dates shown are the means ± SD from three independent experiments. Data are shown from three independent experiments. (**p < 0.01 and ***p < 0.001 compared with Control siRNA plus GEM group).

To investigate the role of HMGB1 in bladder cancer cell's response to GEM, we examined the effect of HMGB1 knockdown on GEM-induced cytotoxicity (Figure 4A), which was associated with increased apoptosis that was detected as increased caspase-3 and PARP cleavage detected by Western blot and Annexin-V positive staining detected by flow cytometry assay (Figure 4B–4D). These results suggest that HMGB1 protects bladder cancer cells against GEM's cytotoxicity.

### HMGB1 knockdown attenuates gemcitabine-induced autophagy

**Figure 5:**
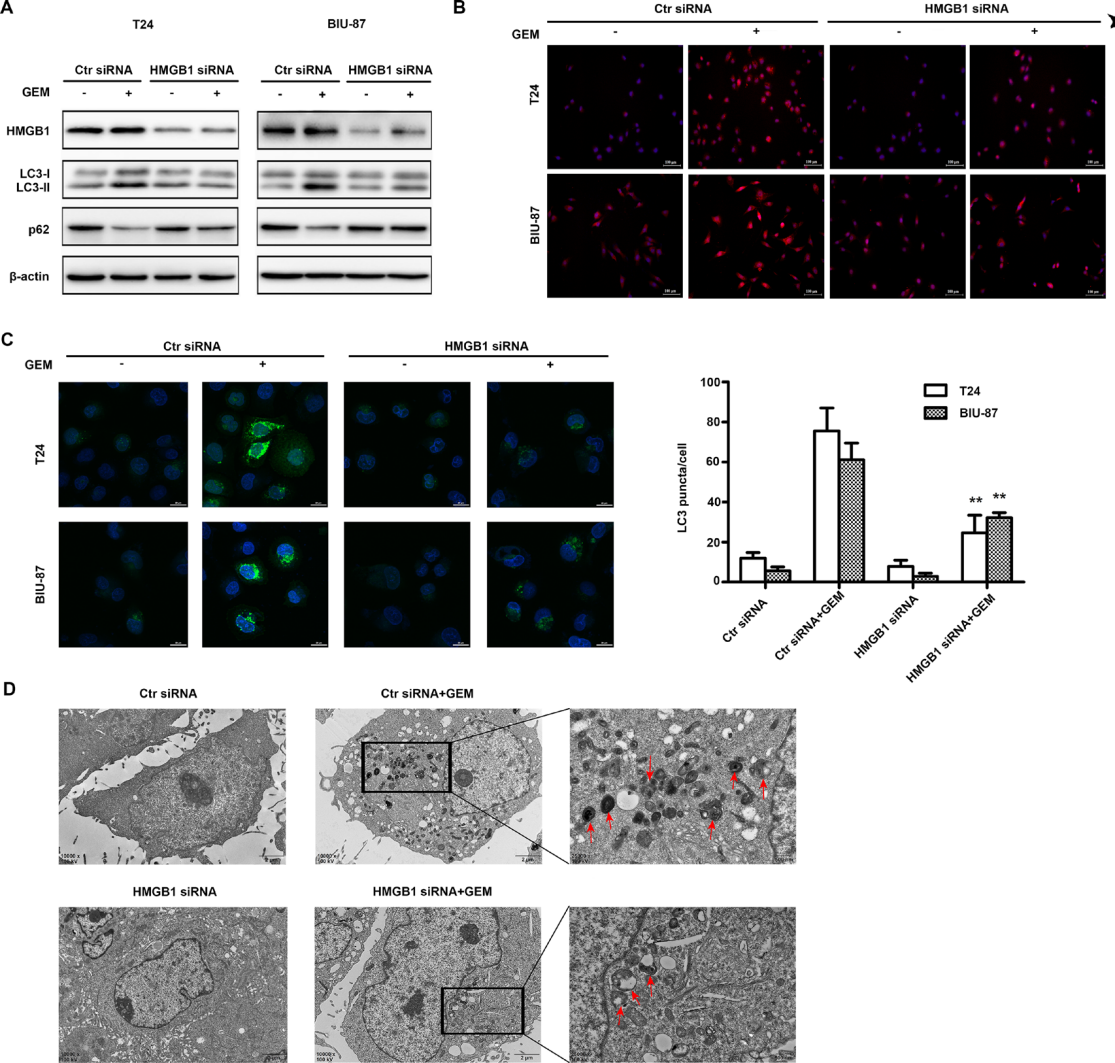
HMGB1 knockdown attenuates GEM-induced autophagy in bladder cancer cells (**A**) The cells were transfected with control siRNA and HMGB1 siRNA for 48 h and dealt with GEM (4 μg/mL) for an additional 8 h, cell lysates were collected for detecting LC3 and p62 by western blotting. (**B**) The treated cells were observed using fluorescence microscopy. Scale Bar = 100μm (Original magnification: ×200). (**C**) The cells were transfected using GFP-LC3-adenoviral vectors and observed using confocal laser microscope. Scale Bar = 20 μm. (**p < 0.01 compared with control siRNA plus GEM group). (**D**) The cells were analyzed by transmission electron microscope. Scale Bar = 2 μm and 0.5 μm, the red arrows showed the characteristic autophagosome.

Previously reports suggested that HMGB1 mediates autophagy induced by chemotherapeutics in other cancer [33]. Thus, we investigated HMGB1 is involved GEM-induced autophagy in bladder cancer cells. Suppressing HMGB1 expression attenuated the LC3-II accumulation and p62 degradation induced by GEM treatment (Figure 5A). GEM-stimulated LC3 puncta formation detected by fluorescence microscopy and confocal microscopy was also suppressed by HMGB1 knockdown (Figure 5B) (Figure 5C). GEM-induced autophgosome formation detected with a transmission electron microscope was also reduced in the HMGB1 knockdown cells (Figure 5D). These results suggest that HMGB1 mediates GEM-induced cell autophagy in bladder cancer cells.

### Blockade of autophagy potentiates gemcitabine's cytotoxicity in bladder cancer cells

**Figure 6:**
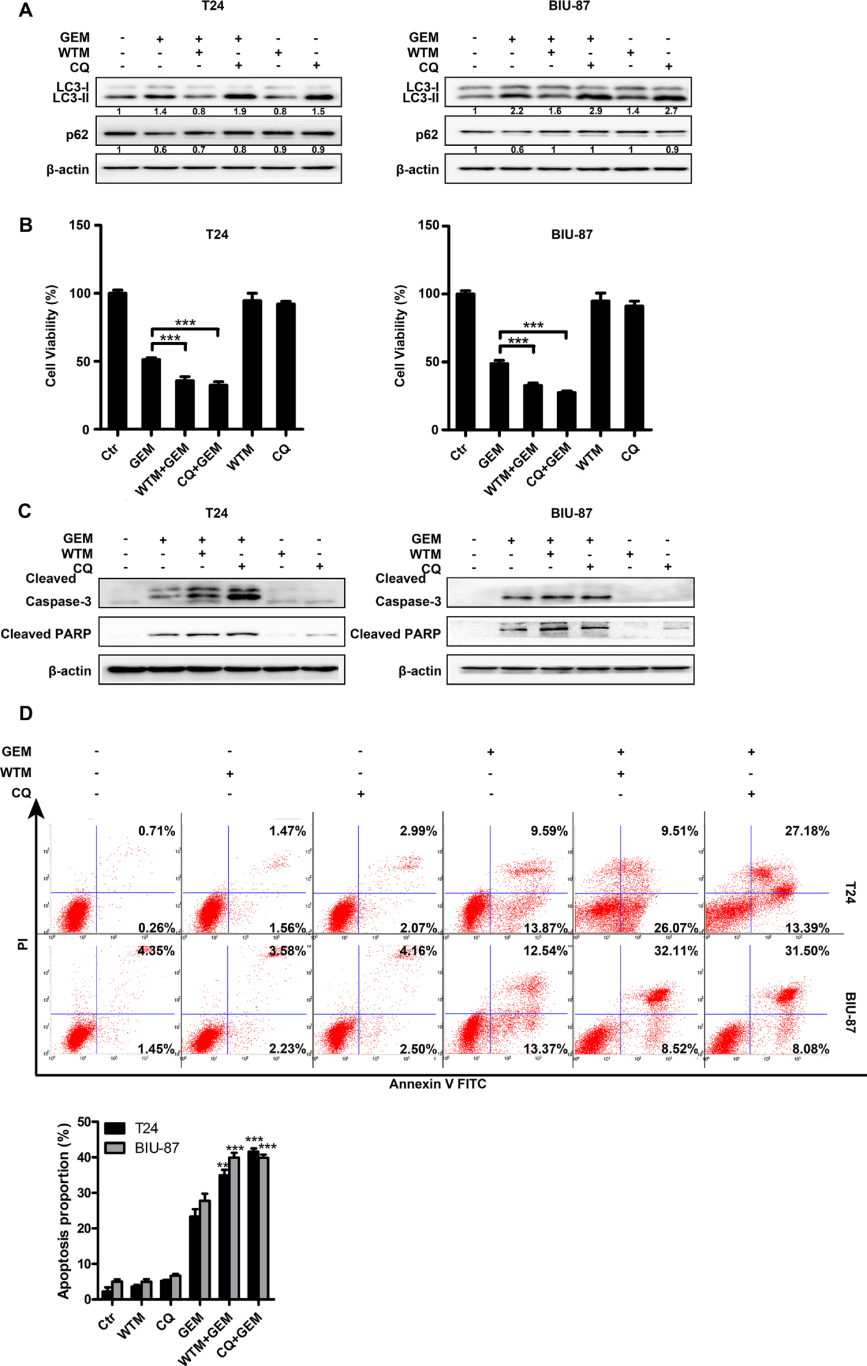
Autophagy protects bladder cancer cells against GEM-induced cytotoxicity (**A**) The cells were pretreated with autophagy inhibitors wortmannin (1μM) and CQ (20 μM) for 30 min and exposed to the indicated concentrations of GEM for another 8 h. The whole-cell lysates were collected for measuring LC3 and p62 by Western blot. (**B**, **C**) The cells were pretreated with the indicated autophagy inhibitors for 30 min and then stimulated by GEM for an additional 24 h. Cytotoxicity was measured using CCK8 assay (B). Cell lysates were subjected to immunoblotting analysis of cleaved caspase-3 and cleaved PARP (C). (**D**) The cells were pretreated with wortmannin (1 μM) and CQ (20 μM) for 30 min and then exposed to the indicated concentration of GEM for 24 h, apoptosis rate was measured by flow cytometry. Dates shown are the means ± SD from three independent experiments. (**p < 0.01 and ***p < 0.001 compared with GEM group).

To determine the role of autophagy in GEM-induced cytotoxicity, we employed two autophagy inhibitors, wortmannin (WTM) and chloroquine (CQ) for blocking autophagy. WTM inhibits autophagy at early stage to attenuate LC3-II accumulation and p62 degradation. CQ suppresses autophagy through inhibiting lysosomal protein degradation, thus, causes additional LC3-II accumulation and reduced p62 degradation. As shown in Figure 6A, compared with GEM alone group, GEM combined with either autophagy inhibitor markedly suppressed GEM-induced autophagy. Suppressing autophagy with WTM and CQ strongly potentiated GEM's inhibitory effect on cell viability (Figure 6B), which was associated with increased caspase-3 and PARP cleavege (Figure 6C) and Annexin-V staining (Figure 6D). Altogether, these results suggest that GEM-mediated autophagy is cytoprotective, and suppressing autophagy can potentiate GEM-induced apoptosis in bladder cancer cells.

### HMGB1 mediates GEM-induced JNK and ERK for autophagy activation in bladder cancer cells

**Figure 7:**
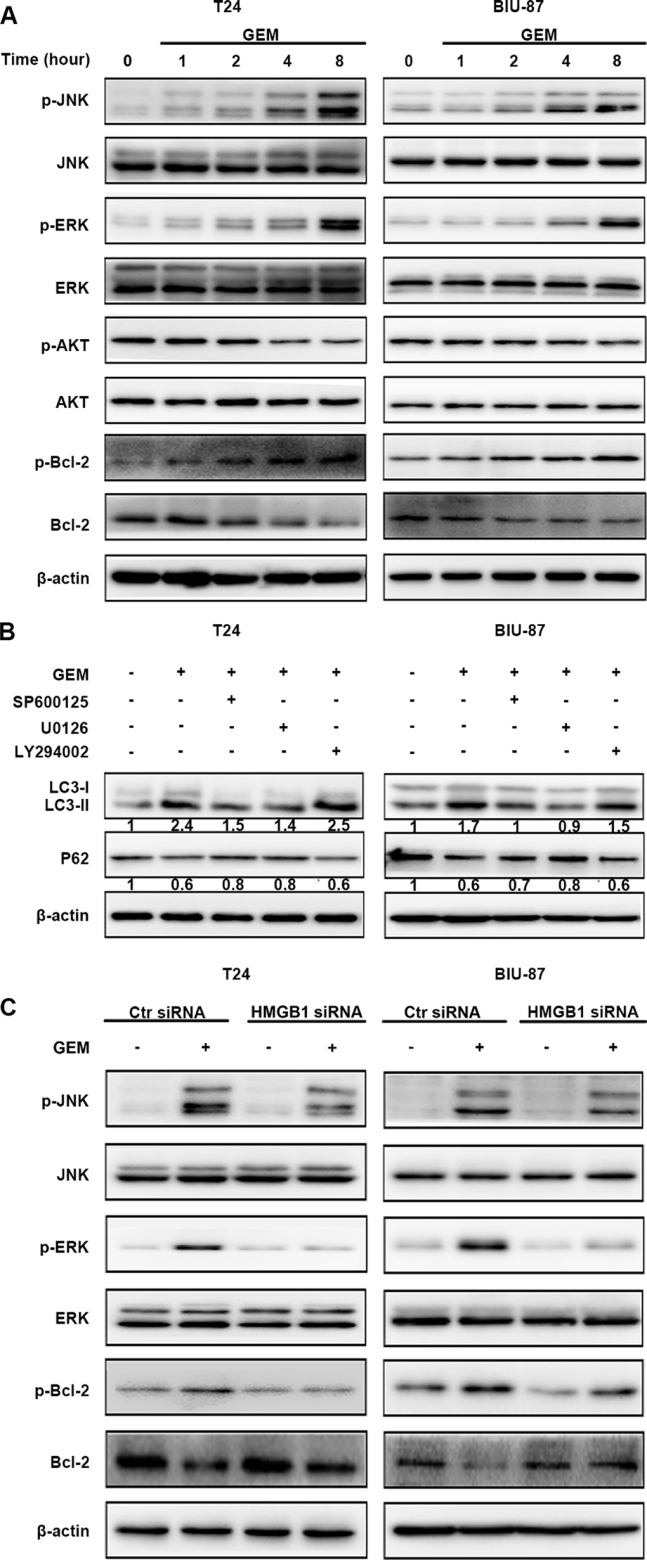
HMGB1 mediates GEM-induced JNK and ERK for autophagy activation in bladder cancer cells (**A**) The cells were treated with 4 μg/mL GEM for indicated times. Then cell lysates were prepared for detecting the indicated proteins by western blotting. (**B**) The cells were pretreated with the indicated inhibitors, SP600125 (10 μM), U0126 (10 μM) or LY294002 (20 μM) for 30 min and incubation with GEM (4 μg/mL) for 8 h. The indicated proteins were detected by western blotting. (**C**) The cells were transfected with control siRNA or a siRNA targeting HMGB1 for 48 h, followed by the treatment of GEM (4 μg/mL) for 8 h. Then whole-cell lysates were prepared for the assay of the indicated proteins by western blotting.

Anticancer treatment can activate multiple signaling pathways, including activate mitogen-activated protein kinase (MAPK) and AKT signal that involved in autophagy induction. We treated the T24 and BIU-87 cells with GEM for different time periods, and examined activation (phosphorylation) of these kinases by Western blot. The results showed that JNK and ERK but not AKT was activated by GEM (Figure 7A). The specific JNK inhibitor SP600125 and ERK inhibitor U0126, but not the AKT inhibitor LY294002, significantly inhibited GEM-induced LC3-II increase and p62 decrease (Figure 7B). HMGB1 knockdown attenuated GEM-induced JNK and ERK activation (Figure 7C). Collectively, these results suggest that HMGB1 mediates GEM-induced JNK and ERK for autophagy activation in bladder cancer cells.

### Blockage of JNK or ERK suppresses Bcl-2 phosphorylation and enhances GEM's cytotoxicity in bladder cancer cells

**Figure 8:**
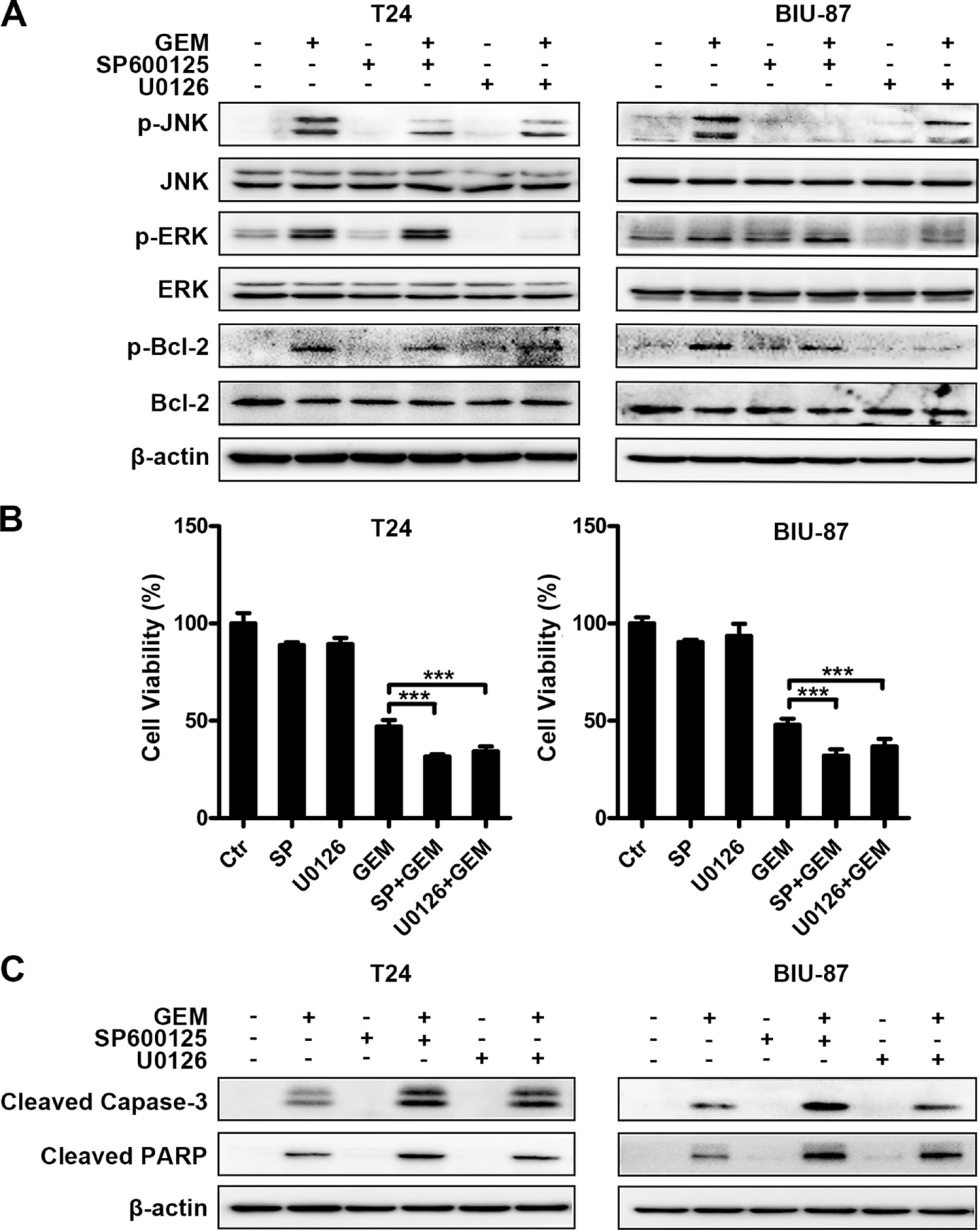
Blockage of JNK or ERK suppresses Bcl-2 phosphorylation and enhances GEM's cytotoxicity in bladder cancer cells (**A**) The cells were pretreated with SP600125 (10 μM) and U0126 (10 μM) for 30 min and incubation with the indicated concentration of GEM for another 4 h. Whole-cell lysates were subjected to western blot analysis for the indicated proteins. (**B**, **C**) The cells were pretreated with the indicated inhibitors for 30 min and exposed to GEM for an additional 24 h. Cell viability was analyzed by CCK8 assay. The results are the representative of at least three identical experiments. (***p < 0.001 compared with GEM group) (B). Whole-cell lysates were collected for analyzing the indicated apoptosis associated proteins by western blotting (C).

Bcl-2 was phosphorylated accompanied with decrease of total Bcl-2 in GEM-treated cells (Figures 7A, 8A). Both SP600125 and U0126 remarkably reduced GEM-induced Bcl-2 phosphorylation (Figure 8A). Suppressing JNK and ERK also increased GEM's effects on cell viability and caspase-3 and PARP cleavage (Figure 8B, 8C). Taken together, all these findings substantiated that JNK and ERK activation are involved in GEM-stimulated Bcl-2 phosphorylation and inhibition of JNK and ERK potentiates GEM-induced apoptotic cytotoxicity in bladder cancer cells.

### Ectopic HMGB1 expression facilitates GEM-induced autophagy and inhibits GEM-induced apoptosis

To strengthen the results derived from HMGB1 knockdown, we examined the effect of ectopic HMGB1 expression on GEM-induced autophagy and apoptosis in T24 cells. Ectopic HMGB1 expression by itself enhanced autophagy induction, which was shown as LC3-II accumulation and p62 degradation (Supplementary Figure 1A). HMGB1 expression increased GEM-induced autophagy and inhibits GEM-induced apoptosis (Supplementary Figure 1B). Together with the results from the HMGB1 knockdown experiments, these data strongly suggest that HMGB1 mediates GEM-induced cytoprotective autophagy to blunt GEM's cytotoxicity in bladder cancer cells.

## DISCUSSION

This study provides evidence showing that HMGB1 mediates autophagy to attenuate GEM-induced apoptotic death in bladder cancer cells, which may play a role in gemcitabine resistance. HMGB1 expression was increased in primary bladder cancer tissue and GEM-treated bladder cancer cells. Suppressing HMGB1 expression strongly potentiated, while ectopic expressing HMGB1 suppressed gemcitabine-induced apoptosis. HMGB1 siRNA or autophagy inhibitors suppressed gemcitabine-induced autophagy. Further, HMGB1 mediates gemcitabine-induced JNK and ERK activation and Bcl-2 phosphorylation. Blocking ERK and JNK inhibited autophagy and increased apoptosis. Thus, our results suggest that while gemcitabine kills bladder cancer cells through apoptosis, it also induces a cytoprotective autophagy involving HMGB1-mediated JNK and ERK activation, and targeting this pathway may improve the anticancer efficacy of gemcitabine against bladder cancer.

The mechanisms of acquired chemoresistance are complex, which involve epigenetic or genetic alterations, detoxifying drugs, and preventing apoptosis, repairing DNA damage and modulating cell cycle in tumor cells, and mechanical or biochemical factors, secretion of cytokines and chemokines in host microenvironment [12–17]. Autophagy alteration may be another contributing factor [18]. Gemcitabine can activate autophagy in different types of cancer cells such as that of bladder cancer [19], breast cancer [20, 21] and pancreatic cancer [22, 23]. The role of autophagy in a cell's fate is cell or tissue type-specific, and depends on stimuli, activation duration and intensity, and related signaling [24, 25]. For example, autophagy is cytoprotective coping with various adverse stimuli for survival of normal cells including mesenchymal stem cells, endothelial progenitor cells and hepatocytes [26–30]. Functioning as a cell survival mechanism, autophagy promotes cancer progression in certain circumstances [31–36]. In contrast, autophagy mediates anticancer therapeutic-induced apoptosis in prostate cancer cells and osteosarcoma cells, and necroptosis in lung cancer and bladder cancer cells [37–39]. In our present study, we found GEM induces autophagy, which was cytoprotective to blunt cytotoxicity of GEM in bladder cancer cells. Thus, blocking autophagy may improve GEM's anticancer activity for bladder cancer therapy.

Previous studies have shown that HMGB1 overexpression is a common and widespread phenomenon in numerous cancers and is involved in cancer progression and malignant behaviors [13, 40–47]. In addition, HMGB1 expression in cancer cells could be increased by a variety of anticancer agents including doxorubicin, cisplatin, methotrexate, docetaxel and gemcitabine [33, 48, 49]. Besides, HMGB1 is passively released from cancer cells or actively secreted by inflammatory cells in response to anticancer treatment. HMGB1 was shown to be involved in autophagy, DNA damage repair and chemoresistance [41, 50]. In this study, we found that HMGB1 expression was significantly increased in bladder cancer tissues, which was associated tumor grade and T stage. HMGB1 expression was induced by GEM, and HMGB1 knockdown promoted GEM's cytotoxicity in bladder cancer cells. Further, we found HMGB1 mediated GEM-induced autophagy. These results suggest that HMGB1 plays a cell survival role through mediating autophagy to inhibit GEM's cytotoxicity in bladder cancer cells.

Emerging evidence shows that MAPK, including JNK, ERK, p38 MAPK, are involved in cell death and autophagy [51–53]. JNK or ERK mediates phosphorylation of Bcl-2 and Bcl-xL, resulting in dissociation of the complex of Bcl-2/Beclin-1 or Bcl-xL/Beclin-1. This process releases Beclin-1 and leads to induction [39, 54]. We found that HMGB1 mediates GEM-induced activation of JNK and ERK. Inhibition of JNK and ERK attenuated Bcl-2 phosphorylation and autophagy activation. Thus, GEM-induced and HMGB1-mediated autophagy is likely through the JNK/ERK/Bcl-2/ Beclin-1 cascade. This is consistent with previous reports showing HMGB1 facilitates the dissociation of Bcl-2/Beclin-1 complex through ERK1/2-mediated Bcl-2 phosphorylation[55], and suppression of HMGB1 resulted in reduction of ERK and Bcl-2 phosphorylation [56]. Other autophagy activation mechanisms may be also involved. For example, HMGB1 potentiates the Beclin-1/PI3K-III complex via the ERK pathway [33].

In summary, our study reveals a complex cellular response to GEM in bladder cancer cells. While GEM kills bladder cancer cells through apoptosis, it also induces a cytoprotective autophagy involving HMGB1-mediated JNK and ERK activation, and targeting this pathway may improve the anticancer efficacy of gemcitabine against bladder cancer.

## MATERIALS AND METHODS

### Reagents and antibody

Antibodies against cleaved caspase-3, cleaved PARP, phospho-JNK, JNK1, phospho-ERK, ERK1/2, phospho-AKT (Ser473),AKT, Bcl-2 and phospho-Bcl-2 were all purchased from Cell Signaling Technology (Cell Signaling Technology, Danvers, MA, USA). Antibody to p62 was purchased from BD Biosciences (New York, USA). Antibody for LC3B was obtained from Sigma Aldrich (St Louis, MO, USA). Antibody for HMGB1 and goat anti-rabbit IgG antibody (Alexa Fluor 594) was obtained from Abcam (Cambridge, MA, USA). Anti-β-actin was purchased from Zoonbio Biotechnology (Nanjing, China). Horse radish peroxidase (HRP) conjugated goat anti rabbit/mouse secondary antibodies were purchased from Abgent (San Diego, CA, USA). The JNK inhibitor SP600125, the ERK inhibitor U0126, the PI3K/AKT inhibitor LY294002, the lysosomal inhibitor chloroquine (CQ) and the autophagy inhibitor wortmannin (WTM) were purchased from Selleckchem (Houston, Texas, USA). Roswell Park Memorial Institute (RPMI)-1640 medium and trypsin were obtained from HyClone (Logan, UT, USA). Fetal bovine serum (FBS) was purchased from Gibco (Thermo Fisher Scientific, MA, USA). Cell viability and cytotoxicity test kits (CCK-8) was purchased from Dojindo Molecular Technologies (Kumamoto, Japan). GFP-LC3 adenoviral vectors were purchase from HanBio Technology (HanBio, shanghai, China). Gemcitabine (GEM) was acquired from the First Affiliated Hospital of Chongqing Medical University. Bladder tumor tissues (consist of 30 paired and 21 non-paired) and adjacent non-cancerous tissues were obtained from patients hospitalized at the first urology ward in the First Affiliated Hospital of Chongqing Medical University.

### Cell lines and cell culture

The human bladder carcinoma cells T24 and BIU-87 were obtained from the Chongqing Key Laboratory of Molecular Oncology and Epigenetics, the First Affiliated Hospital of Chongqing Medical University (Chongqing, China). The cells were cultured in 1640 RPMI medium containing 10% FBS, 100 μg/mL penicillin, 100 μg/mL streptomycin and incubated at 37°C in a humidified atmosphere of 95% air and 5% CO2.

### Cell viability

Cells were seeded into 96-well plates at the density of 5 × 10^3^ cells with 100 μl medium per well and cultured for 24 h, then treated with the required drugs and reagents for another 24 h, and 10 ul of CCK8 was added into each well. The cells were incubated for an additional 4 h at 37°C. Absorbance (450 nm) was measured using a Tecan Infinite F200/M200 type multifunction microplate reader (Tecan, Männedorf, Switzerland). The cell viability was calculated as the following method: Cell viability (%) = (the average OD value of experimental group/the average OD value of control group) ×100%.

### Western blot analysis

To get the whole-cell lysates, cells were treated for various periods, washed with cold PBS for three times, lysed with RIPA lysis buffer (Beyotime, Haimen, China) and 1% PMSF (Beyotime, Haimen, China) for 30 min at 4°C, then centrifuged at 12000 × g for 15 min at 4°C. Concentration of protein was measured by a bicinchoninic acid (BCA) kit (Beyotime, Haimen, China). The prepared protein samples were loaded and subjected to 12% SDS-polyacrylamide gel electrophoresis. PVDF membranes (Merck Millipore, Darmstadt, Germany) were used for transfer. The target bands were blocked with 5% skim milk for 1 h and then incubated with the different primary antibodies overnight at 4°C. After being washed by TBST (10mmol/L Tris, pH 7.5; 100 mmol/L NaCl; and 0.1% Tween20), the bands were incubated with the goat anti- rabbit/mouse secondary antibody labeled with horseradish peroxidase for 1 h at room temperature. Later, the blots were detected using electrochemiluminescence assay.

### Transfection and confocal microscopy

Cells were transfected with HMGB siRNA (Sense: GGAAGUUUCUACUGUAUAGTT; Antisense: CUAUACAGUAGAAACUUCCTT)or control siRNA (Sense: UUCUCCGAACGUGUCACGUTT; Antisense: ACGUGACACGUUCGGAGAATT) (Sangon Biotech, Shanghai, China) using lipofectamine2000 (Invitrogen, USA) and Opti-MEM (Invitrogen, USA) according to the manufacturer's protocol, then transfected with adenoviral vectors carrying GFP-LC3for 24 h, treated with GEM for indicated time, fixed in 4% formaldehyde for 10 min, wash with PBS, stained with DAPI for 10 min. The samples were examined under a confocal laser scanning microscope (Olympus Fluoview 2000). The GFP-LC3 puncta in each cell were quantified by Image J software. Transfections with pcDNA3.1 control or pcDNA3.1 HMGB1 (GenePharma, shanghai, China) were performed as mentioned above.

### Apoptosis assays

Cells were treated by the required drugs and reagents for the indicated periods, trypsinized, washed, collected and re-suspended in binding buffer, stained with Annexin V-FITC and propidium iodide (PI; Beyotime, Haimen, China) and detected by flow cytometric (BD Biosciences, San Jose, CA, USA) analysis.

### Immunofluorescence

The treated cells were fixed in 4% formaldehyde for 10 min and permeabilized with 0.1% Triton X-100 (Sigma, USA) for 15 min at room temperature, then labelled with a specific primary antibody at 4°C overnight, rinsed in PBS, treated with the goat anti-rabbit IgG (H +L) antibody labelled with Alexa Fluor 594 for 1 h at room temperature, washed with PBS, stained with DAPI for 10 min, washed with PBS. Finally, the cells were observed by fluorescence microscope.

### Immunohistochemistry

Formalin-fixed and paraffin-embedded tissue sections were dewaxed with xylene and rehydrated with ethanol, immersed in 0.01 mol/L citrate buffer (PH 6.0) for 30min in water bath at 96°C for antigen retrieval, blocked with 3% hydrogen peroxide to remove endogenous peroxidase activity for 10 min, blocked with 10% goat serum for 15 min at room temperature, incubated with anti-HMGB1 in humidified chamber overnight at 4°C, washed with PBS for three times, incubated with the HRP conjugated secondary antibody for 20min at room temperature. Then the slides were stained by the use of DAB (ZSGB-BIO, Beijing, China) for about 1 min and counterstained using hematoxylin for 10 s. The negative control samples were incubated with PBS instead of anti-HMGB1. The score criterion according to the staining intensity and extent was described in previous study [11].

### Transmission electron microscopy

The cells were fixed with 2.5% glutaraldehyde and 1% osmic acid, dehydrated, embedded, solidified, sliced, then the sections were stained with uranyl acetate-lead citrate for observation under transmission electron microscopy (Hitachi-7500, Tokyo, Japan). Digital images were obtained using a NIS-Elements Viewer 4.20.

### Statistical analysis

All statistical analyses are performed using SPSS 19.0 (SPSS Inc., Chicago, USA). Data are expressed as the mean ± standard deviation (SD) from at least three independent experiments. One-way analysis of variance (ANOVA) is used to analyze statistical comparisons. The immunohistochemical results are evaluated by Chi-square test. For all analyses, *p* < 0.05 is considered statistically significant.

## SUPPLEMENTARY FIGURE


