# Supplementary material for: HMGB1-mediated autophagy attenuates gemcitabine-induced apoptosis in bladder cancer cells involving JNK and ERK activation
Source: Oncotarget. 2017 May 11;8(42):71642–56. doi: 10.18632/oncotarget.17796 (PMC5641078; doi:10.18632/oncotarget.17796)
Supplement: Supplementary file 1 [file oncotarget-08-71642-s001.pdf]

HMGB1-mediated autophagy attenuates gemcitabine-induced apoptosis in bladder cancer cells involving JNK and ERK activation

Supplementary Materials

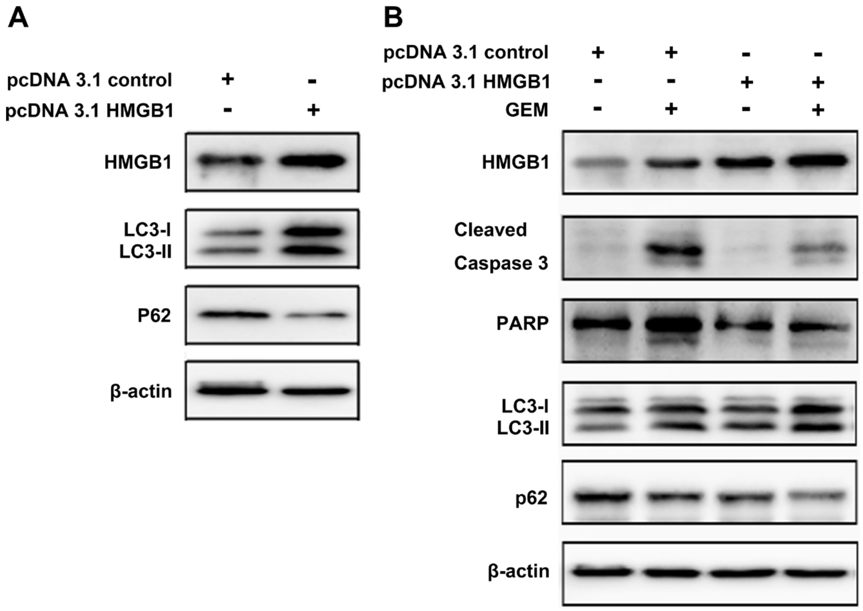

Supplementary Figure 1: Ectopic HMGB1 expression facilitates GEM-induced autophagy and inhibits GEM-induced apoptosis. (**A**) T24 cells were transfected with pcDNA3.1 control or pcDNA3.1 HMGB1 for 48 h. Cell lysates were subjected to western blotting analysis for LC3, p62 and HMGB1. (**B**) T24 cells transfected with pcDNA3.1 control or pcDNA3.1 HMGB1 for 48 h were treated with GEM (4 μg/mL) for further 12 h. Cell lysates were collected for analyzing the proteins of LC3, p62, HMGB1, cleaved caspase-3 and PARP by western blotting.
